# Stakeholder Perspectives of Attributes and Features of Context Relevant to Knowledge Translation in Health Settings: A Multi-Country Analysis

**DOI:** 10.34172/ijhpm.2021.32

**Published:** 2021-05-01

**Authors:** Janet E. Squires, Alison M. Hutchinson, Mary Coughlin, Kainat Bashir, Janet Curran, Jeremy M. Grimshaw, Kristin Dorrance, Laura Aloisio, Jamie Brehaut, Jill J. Francis, Noah Ivers, John Lavis, Susan Michie, Michael Hillmer, Thomas Noseworthy, Jocelyn Vine, Ian D. Graham

**Affiliations:** ^1^Department of Health Sciences, School of Nursing, University of Ottawa, Ottawa, ON, Canada.; ^2^Clinical Epidemiology Program, Ottawa Hospital Research Institute, Ottawa, ON, Canada.; ^3^Monash Health, Melbourne, VIC, Australia.; ^4^School of Nursing and Midwifery, Centre for Quality and Patient Safety Research, Institute for Health Transformation, Deakin University, Geelong, VIC, Australia.; ^5^Institute of Health Policy, Management and Evaluation, University of Toronto, Toronto, ON, Canada.; ^6^Faculty of Health, School of Nursing, Dalhousie University, Halifax, NS, Canada.; ^7^IWK Health Centre, Halifax, NS, Canada.; ^8^Faculty of Medicine, University of Ottawa, Ottawa, ON, Canada.; ^9^Statistics Canada, Ottawa, ON, Canada.; ^10^School of Epidemiology and Public Health, University of Ottawa, Ottawa, ON, Canada.; ^11^School of Health Sciences, University of Melbourne, Melbourne, VIC, Australia.; ^12^Women’s College Hospital, Toronto, ON, Canada.; ^13^McMaster University, Hamilton, ON, Canada.; ^14^University College London, London, UK.; ^15^Ontario Ministry of Health and Long-Term Care, Toronto, ON, Canada.; ^16^University of Calgary, Calgary, AB, Canada.

**Keywords:** Context, Knowledge Translation, Implementation

## Abstract

**Background:** Context is recognized as important to successful knowledge translation (KT) in health settings. What is meant by context, however, is poorly understood. The purpose of the current study was to elicit tacit knowledge about what is perceived to constitute context by conducting interviews with a variety of health system stakeholders internationally so as to compile a comprehensive list of contextual attributes and their features relevant to KT in healthcare.

**Methods:** A descriptive qualitative study design was used. Semi-structured interviews were conducted with health system stakeholders (change agents/KT specialists and KT researchers) in four countries: Australia, Canada, the United Kingdom, and the United States. Interview transcripts were analyzed using inductive thematic content analysis in four steps: (1) selection of utterances describing context, (2) coding of features of context, (3) categorizing of features into attributes of context, (4) comparison of attributes and features by: country, KT experience, and role.

**Results:** A total of 39 interviews were conducted. We identified 66 unique features of context, categorized into 16 attributes. One attribute, Facility Characteristics, was not represented in previously published KT frameworks. We found instances of all 16 attributes in the interviews irrespective of country, level of experience with KT, and primary role (change agent/KT specialist vs. KT researcher), revealing robustness and transferability of the attributes identified. We also identified 30 new context features (across 13 of the 16 attributes).

**Conclusion:** The findings from this study represent an important advancement in the KT field; we provide much needed conceptual clarity in context, which is essential to the development of common assessment tools to measure context to determine which context attributes and features are more or less important in different contexts for improving KT success.

## Background

**Key Messages**

**Implications for policy makers**

- Context is important to knowledge translation (KT) success in health settings and should be considered when planning KT initiatives.
- The 16 attributes of context identified in this study were consistent across stakeholders, irrespective of the stakeholder’s country, years of KT experience and primary role, supporting broad utility of the attributes of context identified.
- The attributes of context reported should be considered when designing, implementing and evaluating KT initiatives.

**Implications for the public**

Evidence shows that healthcare professionals’ use of research evidence in their practice is not optimal. The context of healthcare influences use of research by healthcare professionals. What is meant by context, however, is poorly understood and needs to be answered in order to guide the planning and delivery of healthcare to promote research use. This paper reports the findings of interviews with a variety of health system stakeholders, internationally, to identify a list of context attributes and their features relevant to KT in health settings. In total, 66 features of context were identified by stakeholders and these were categorized into 16 broader attributes of context. The attributes and features were consistent, across the stakeholders’ countries, level of KT experience, and primary role (change agent/KT specialist vs. KT researcher), revealing robustness and transferability of our attributes of context.

Internationally, healthcare professionals’ use of research evidence in clinical practice is suboptimal, despite increased awareness of evidence and known gaps in its use.^1-8^ In response, there is widespread interest in the field of knowledge translation (KT)/implementation. The KT field comprises KT science (scientific study of methods to promote the uptake of research findings into routine healthcare in clinical, organizational or policy contexts)^9^ and KT practice (act of applying advancements from KT science in practice; for example, using implementation strategies of known effectiveness to support people, organizations, and/or systems to use evidence in decision-making).^10^ Researchers have identified several major conceptual and methodological issues facing the KT field; among them is the need to assess and report on the influence of context in KT studies.^11-30^ This first requires conceptual clarity of context and particularly, what comprises context.

 It is unclear what is meant by ‘context’ or even if authors are referring to the same concept when they use the term. For example, Ovretveit^31^ and Rycroft-Malone^32^ define context broadly. Ovretveit^31^ defines context as all factors that are not part of the intervention, and Rycroft-Malone^32^ defines it as “the environment or setting in which the proposed change is to be implemented” (p. 299). May et al,^33^ on the other hand, defines context more specifically, as “the physical, organizational, institutional, and legislative structures that enable and constrain, and resource and realize, people and procedures.” In addition to multiple definitions, context has been examined in implementation research from a large variety of perspectives including syntheses on the determinants of innovation adoption,^11,34,35^ the role of context in quality improvement,^29,30,36,37^ context features associated with research utilization^38-41^; as well as its role in KT frameworks ^(eg,42-51)^ and in the development of instruments to measure context ^(eg,23,41,51-55)^. While each of these reports suggests that context is vital to KT in health settings, there is little agreement across them on what comprises context and thus, which attributes and features of context are important to assess to optimize KT in the health sector.

 Several KT frameworks exist that recognize the importance of context. However, they provide little detail on what are the key attributes and features of context. Rogers’ Diffusion of InnovationsTheory,^56^ first published in 1962, is a commonly used theory in KT studies. It equates innovation adoption with evidence implementation and describes it as being related to a variety of contextual features such as leadership, centralization, complexity, formalization, interconnectedness, and organizational slack. In 1998, Kitson and colleagues^48^ developed the *Promoting Action on Research in Health Services ( PARiHS ) *framework. In this framework, successful KT is hypothesized to be a function of evidence, context (defined as leadership, culture, and feedback), and facilitation.^32,44,57^ The *PARiHS* framework was revised in 2016 and termed the *Integrated* - *Promoting Action on Research in Health Services ( i-PARiHS ).*^58^ The context construct was expanded on in the revised framework, differentiating inner (including both the immediate local setting (eg, unit, hospital department or primary care team) and the organisation within which this unit or team is embedded) from outer context (the wider health system in which the organisation is based and reflects the policy, social, regulatory and political infrastructures). In 2002, Champagne^59^ proposed a framework of factors to consider when producing change in organizations including context: the implementation climate (incentives, physical, human, and cognitive resources; change management strategies) and structural characteristics (organicity, complexity, and integration). The *Knowledge to Action Framework,*^60^ published in 2006, also highlights the importance of context to successful KT. Included in this framework are processes related to context necessary to implement evidence in healthcare, namely: adapting evidence to the local context and the assessing the barriers and enablers (which include context) to evidence use. In 2009, Damschroder et al^38^ published the Consolidated Framework for Implementation Research (CFIR). This framework includes eight concepts related to the intervention itself, four related to the outer context (eg, the patient and resources), twelve related to the inner setting or context (eg, culture and leadership), five related to the individual, and eight related to processes (such as planning and reflection). Context as conceptualised in CFIR is reflective of organizational literature, where context is examined from the perspective of levels. In the organizational literature, three levels of context are commonly proposed: macro in which market-type forces are at play, meso in which organizational characteristics are an influence; and micro in which activities in the clinical setting provides a contextual influence.^61^ More recently, in 2013, Flottorp and colleagues^50^ published the Tailored Implementation in Chronic Diseases (TICD) checklist, which draws on a consolidation of KT frameworks that include context. This checklist contains 7 broad domains, of which 5 are related to context: patient factors, professional interactions, incentives and resources, capacity for organizational change, and social, political and legal factors.

 Despite the existence of many KT theories and frameworks that include context, as illustrated above, when examined closely several issues are evident. First, while each theory/framework includes context, they are limited in the number of attributes and features of context they identify. In our previous research in this area, we identified several features of context that are not captured in commonly used KT frameworks: TICD, CFIR and PARiHS/i-PARiHS. These include: patient population features (eg, patient demographics), healthcare provider features (eg, job autonomy, level of experience), work structure features (eg, workload, continuity of care), and facility features (eg, type of facility, size of facility).^61^ Second, these theories/frameworks are inconsistent with each other regarding the attributes and features of context they include; some features and attributes are represented in more than one framework (eg, leadership in TICD, CFIR, PARiHS/i-PARiHS), while others are unique to a single framework (eg, continuing educational system in TICD but not in CFIR, PARiHS/i-PARiHS). Further, no two frameworks contain the exact same set of context features and attributes. This lack of correspondence between frameworks raises questions about the comprehensiveness of existing frameworks with respect to context. Third, health system stakeholders, defined as individuals responsible for the design and implementation of KT strategies, programs, and change processes focused on improving healthcare professionals’ use of evidence in health settings, have seldom been engaged in developing these theories/frameworks. Health system stakeholders include change agents and KT specialists (eg, healthcare decision-makers, knowledge brokers, implementation practitioners, quality improvement specialists) and KT researchers. Such individuals have tacit firsthand knowledge of KT and the effects of context on KT that have not yet been systematically explored. Hence, their knowledge on what attributes and features of context are important to research use by healthcare professionals is necessary to further elucidate the concept of context. As a result, the purpose of this study was to conduct interviews with a variety of health system stakeholders internationally in order to compile a comprehensive list of contextual attributes and their features relevant to KT in health settings. Specifically, we aimed to identify contextual features perceived by stakeholders to: (1) facilitate or hinder healthcare professionals’ use of research in clinical practice settings; and (2) facilitate or hinder KT strategies to improve healthcare professionals’ use of research evidence in clinical practice settings.

## Methods

###  Study Design and Sample

 A descriptive qualitative study design was used. The sample consisted of health system stakeholders, defined as individuals who are responsible for the design and/or implementation of interventions, programs, and change processes focused on improving healthcare professionals’ use of research evidence in clinical practice settings (eg, hospitals, primary care). This group includes both change agents (eg, KT and quality improvement specialists) and researchers who are undertaking KT work (hereafter referred to as KT researchers). Participants were purposefully selected from the three Canadian provinces where our research team’s change agent investigators resided (Nova Scotia, Ontario, Alberta) as well as in Australia, United Kingdom, and United States, where our international researcher investigators resided. A purposive list of potential participants from each country was developed. A maximum variation approach across countries was used to optimize diversity in primary role and experience in KT to yield a range of perspectives. All research team members created a list of potential participants (by role – change agents/KT specialists and KT researchers) for their country (in the case of Canada, for their province) by drawing on their extensive professional networks. These lists were then merged by country so that eight lists resulted, two for each country (one for change agents/KT specialists and one for KT researchers). Snowball sampling^62^ (asking recruited individuals to recommend additional participants) was also used in each country. Potential participants were contacted by email. Participants were purposefully selected from each country to ensure we interviewed participants with varying levels of experience (less than 5 years and 5 or more years) in KT and varying roles (change agents/KT specialists and KT researchers). Sample size was informed by the concept of data saturation, interviews were conducted in each country until no new information was offered, meaning no new codes were obtained in the final interview of each country.^63^ Broadly informed by previous research,^64-66^ a minimum of five participants per country was estimated to be needed to achieve data saturation.

###  Data Collection and Analysis

 Semi-structured one-on-one interviews were conducted in person or by phone over a 16-week period (May-August 2016). Interviewers and participants (called key informants) had no prior relationships before study commencement. Key informants were made aware of the goals of the research study prior to the interviews. Interview questions were open-ended and designed to elicit key informants’ tacit knowledge about context (what features did they perceive to comprise context which are relevant to KT in health settings). We did not present a definition of context to our key informants as we wanted to elicit their perception of what comprises context – we asked each key informant to define context, what ‘context’ meant to them, and what features of context were relevant to KT in health settings. Probes were used throughout the interviews, where appropriate, to explore specific features of context identified by the key informant in more detail and to ensure clarity. The interview guide was pilot-tested with three Canadian stakeholders (two hospital-based change agents – one KT specialist and one quality improvement specialist) and one KT researcher. The pilot-test participants were not part of the larger study sample. Based on the pilot interviews, a short series of background questions were added to the interview guide (eg, Can you give me a brief overview of your professional role including previous relevant roles/responsibilities? What education or training have you completed to prepare you for your work?). No other modifications to the interview guide were made.

 Interviews were approximately 30 minutes in duration, digitally recorded, transcribed verbatim and verified by the interviewer prior to analysis. Transcripts were not returned to key informants to be verified. The transcripts were imported into NVivo 10 software.^67^ Data were analyzed independently by research team members trained in qualitative analysis (JES, MC, AH, KB, KD, IDG) using inductive qualitative thematic content analysis.^68,69^ We used an inductive, rather than deductive, analytic approach because existing context frameworks are limited in the features and attributes of context that they identify.^61^ Thus, a deductive approach would have restricted our lens, leading to potentially missed features and attributes of context. Our inductive analysis occurred in four systematic steps: (1) selection of utterances relating to context, (2) coding of features of context, (3) categorizing of features of context into higher level attributes of context, and (4) comparison of attributes and features by: (*i*) country (was the code present in interviews from multiple countries), (*ii*) level of KT experience (was the code present in interviews with key informants with more and less KT experience, using five years of experience as the cut point for more vs. less), and (*iii*) primary role (was the code present in both change agents/KT specialists and KT researchers). Analysis by country allowed us to assess whether the contextual attributes and their features identified were likely transferrable to diverse settings. Analysis by KT experience was conducted to see if more stakeholders with more experience with KT, based on greater tacit knowledge, would identify additional attributes and features of context, beyond those identified by stakeholders with less KT experience. Five years was chosen as the cut-off based on past research. According to Benner’s^70^ Novice to Expert Model, in the acquisition and development of skills, nurses pass through five levels of proficiency: novice, advanced beginner, competent, proficient, and expert. The dividing point between beginners (novice, advanced beginner, competent) and experts (proficient, and expert) is estimated to be between 3 and 5 years.^70^ Accordingly, we classified stakeholders with 0-5 years of KT experience as ‘less’ experienced and those with greater than 5 years of KT experience as ‘more’ experienced. Finally, with respect to primary role, change agents/KT specialists have first-hand tacit knowledge of KT and the effects of context on KT. Hence, we wanted to see if they would identify additional attributes and features of context that are relevant to KT beyond those identified by KT researchers.

 Each transcript was first read and key ideas (ie, utterances) that reflected context were highlighted independently by two research team members. These context utterances were assigned a ‘code’ and labelled as a feature of context. Codes (features) were operationally defined in order to be consistently applied throughout the data. Following coding of all interviews, theoretical definitions were developed for each feature by reviewing all data coded to the feature. This was completed by three team members independently, followed by revision and consensus on the definitions. Two team members then merged similar codes (features of context) to create broader categories (attributes of context), which became our main units of analysis in this study. Each category (attribute) of context identified in the transcripts was given a label, definition, and guideline for identification. Theoretical definitions for each attribute were developed by reviewing all features and their definitions assigned to the attribute. This was completed by three team members independently. The team members responsible for this process met and compared their interpretations bi-weekly and jointly selected a label that best represented the category and a definition. All disagreements in the coding, categorizing and definitional phases were discussed and consensus sought. Finally, attributes of context and their features were examined for their presence (or not) by: (1) country (Canada, United States, United Kingdom, and Australia); (2) more (>5 years) or less (0-5 years) experience in KT; and (3) primary role (change agent/KT specialist and KT researcher). Throughout data analysis, codes (features of context) and categories (attributes of context) were discussed and refined within the research team until agreement was reached.^71^

 Rigor was established following criteria set forth by Lincoln and Guba.^72^ Credibility (to establish confidence that our results, from the perspective of the stakeholders we interviewed, are true, credible and believable^72^) was established through examining data across multiple countries, having multiple team members discuss the findings and generate and reach consensus on the key categories (attributes of context),^73^ and through an audit trail. The audit trail was maintained by documenting discussions and decisions made throughout data collection and analysis. Dependability (to ensure our findings are repeatable if the study occurred in the same cohort of key informants, coders and context^72^) was established by providing a rich description of our study methods and an audit trail. Confirmability (to extend the confidence that our results can be confirmed or collaborated by other researchers^72^) was established through reflexivity (we implemented regular team analytic meetings which included senior KT stakeholders, where we reflected on our findings) and triangulation (multiple research team members were involved in coding the data from multiple groups of stakeholders (change agents/KT specialists and KT researchers) across four countries). Finally, transferability (to extend the degree to which the results can be transferred to other contexts or settings^72^) was established through purposeful sampling (by experience and role across countries) and data saturation.

## Results

###  Sample Characteristics

 A total of 91 potential key informants were approached, of which 39 agreed to be interviewed for a response rate of 43%. The sample was drawn from four countries, Australia (n = 12), Canada (n = 14), the United Kingdom (n = 6), and the United States (n = 7). The majority of key informants were female (n = 27), worked within a public healthcare model (n = 33) and had greater than 10 years of experience in KT (practice or research) (mean 11.9 years). There was equal distribution in terms of the key informants’ self-identified primary role (employed in a health system as a change agent/KT specialist, n = 19 or as a KT researcher in a higher education setting or research institute, n = 20). Table 1 summarizes the sample’s characteristics.

**Table 1:** Key Informant Demographics (N = 39)

**Characteristic**	**n **	**Characteristic**	**Mean (Range)**
Country		Years of experience in current role	5.0 (<1-24)
Australia	12		
Canada	24		
United Kingdom	6		
United States	7		
Gender		Years of experience in current organization	11.7 (1.5-39)
Male	12		
Female	27		
Model of care		Years of experience in KT	11.9 (1.5-36)
Public	33		
Private	6		
Primary professional role			
Agent/KT specialist	19		
KT researcher	20		

Abbreviation: KT, knowledge translation.

###  Complexity and Interrelatedness of Contextual Attributes

 While the focus of this study was to identify specific attributes of context and their features that are relevant to KT from the perspective of health system stakeholders, key informants frequently discussed multiple attributes and features simultaneously, illustrating the complexity and interrelatedness of context. To illustrate, we include a quote from a participant from each country, in which they describe different and multiple facets of context.

 A participant from Australia refers to context as varying by a range of factors, including jurisdictional policy, geography, health professional groups, the patient population and organisational culture.

*“…there can be different policies depending on the jurisdiction that you’re in. Also in terms of geography, whether you’re talking about an urban or rural or a remote location. Within stakeholders, I guess I’m thinking not just about clinicians, who your clinicians, who your health professionals are, but who your patient population is. And also I would put in things like culture, organizational culture. I think that’s a really important part of context as well. I’m pretty sure there is more” *(Australia, Public, Participant 2).

 A Canadian participant similarly identifies multiple aspects of context: the organisational culture, staff mix and patient population, in addition to the physical environment.

*“ So I, I context can mean different things like I sort of categorize it in terms of the organizational culture. The context of the organizational culture the physical environment, the clinical makeup of the staff as well as the clinical makeup of the patients” *(Canada, Public, Participant 3).

 Identifying different aspects of context, a participant from the United Kingdom illustrated the complexity of context when referring to potential research users’ resistance to change and concerns about shifts in power.

*“So, you know, it’s hard to say whether people who say ‘Well the evidence isn’t strong enough.’ If they’re actually making that purely rational decision or whether that’s just their, their rationalization or their story for opposition, which maybe have some other psychological cause let’s say. Like they don’t like it because it’s to do with power and this change particularly putting in place this kind of system shifts power away from clinicians to patients. And no clinician is gonna stand up and say ‘I don’t think patients should be empowered.’ But they might stand up and say ‘I don’t think the evidence is strong enough we don’t want to do this’” *(United Kingdom, Public, Participant 4).

 From the United States, a participant refers to context as comprising communication networks, access to resources to guide and support implementation, and accountability as part of shared governance.

*“And for that they, you know, everybody was—all the hospitals have a chief nurse and the chief nurses get together once/month to talk with the leader of the nurse core who’s a general officer. So that’s one mechanism to get the word out on how is this being implemented and how are you doing? There were implementation guides. There were training manuals. So for example, to do shared governance they didn’t call it governance they called it accountability because it’s not just governance for your unit” *(United States, Public, Participant 3).

 These quotes illustrate participants’ perceptions of multiple facets of context and that each facet cannot be considered in isolation. Considering these four quotes collectively also highlights that context is likely complex because people perceive it in different ways.

###  Specific Context Attributes and their Features 

####  Overview

 In total, 66 unique *features of context* perceived to be relevant to KT in health emerged from the 39 interviews with health system stakeholders. All features surfaced in at least two interviews. We grouped the 66 unique features into 16 broader *attributes of context*: (1) Patient Characteristics, (2) Health Professional Characteristics, (3) Collaboration, (4) Culture, (5) Evaluation, (6) Facility Characteristics, (7) Financial Considerations, (8) Governance, (9) Leadership, (10) Organizational Readiness for Change, (11) Professional Role, (12) Resource Access, (13) System Features, (14) Work Structure, (15) Political Climate, and (16) Regulatory and Legislative Standards. The number of features in each attribute varied from 1 (in the *Culture *and* Political Climate* attributes) to 11 (in the *Resource Access* attribute). Table 2 contains a listing of the 16 attributes of context and a consensus definition for each attribute derived by the research team. Table 3 contains a list of the 66 features identified, organized by attribute, and an illustrative quote for each feature. Tables 4 and 5 summarize the presence of the context attributes and features by country, and level of KT experience and primary role, respectively.

**Table 2:** Context Attributes (n = 16) and Definitions

**Attribute**	**Definition**	**Attribute**	**Definition**
Patient characteristics	Patients are the individuals receiving services. This attribute reflects the characteristics of patients/clients/consumers when considered as a group, rather than as individuals, thus all features considered for inclusion here have to be generalizable to a patient population.	Leadership	Leadership refers to the types and styles of leaders within an organization.
Health professional characteristics	The characteristics, expertise and behaviour of the individuals working as providers of services. This attribute reflects individuals when considered as a group rather than as individuals, thus all features considered for inclusion here have to be generalizable to a service provider population.	Organizational readiness for change	The organizational members' shared resolve to implement a change.
Collaboration	To work jointly with others (including other organizations) or together.	Professional role	Expectations, both formal and informal, associated with a given healthcare occupation.
Culture	The inherited ideas, beliefs, values, and attitudes of a group.	Resource access	Access to resources of any kind.
Evaluation	The systematic collection of information about the activities, characteristics, and outcomes of programs, services, policies, or processes in an organization, in order to make judgements about the program/process, improve effectiveness, and/or inform decisions about future development in that organization.	System features	Distinct characteristics of a group of related parts that move or work together in order for a healthcare region, organization, hospital or clinical practice to run effectively.
Facility characteristics	This attribute reflects the characteristics of facilities within an organization. This includes features such as geography, type of facility, and size of the facility.	Work structure	The arrangement of tasks, responsibilities, and resources within and between the various teams working in a clinical setting.
Financial considerations	The income and expenditures relating to service delivery within organizations.	Political climate	The aggregate, current mood and opinions of a populace about political issues that also currently affect that population, in a general sense.
Governance	Therules, policies, systems, structures and processes by which an organization is organised, controlled and directed.	Regulatory and legislative standards	Statutes or principles established and enforced by an agency external to the health professions. Regulatory or legislative standards are here distinguished from guidelines insofar as these standards are binding, often based on law or remuneration structures, and are outside the control of health organizations.

**Table 3:** Context Features (n = 66) and Illustrative Quotes for Each Feature

**Feature**	**Illustrative Quote**
**Context Attribute: Patient Characteristics**	
Patient demographics	* “It probably will take a bit longer in the population that we’re interested in because they’ll be a lot of non-English speaking patients” *(Australia, Public, Participant 2).
Patient expectations and preferences	*“And I think patient needs or something to do with patients or the public is always, always has to be an important consideration and it’s liable to, well I guess the difference is whether you include that and for all these things actually is whether you consider this to be part of context or part of something else. But things like patient perspective never go away and always have to be addressed”* (United Kingdom, Public, Participant 4).
**Context Attribute: Health Professional Characteristics**	
Group composition	*“ So we wanted it to be a whole team approach, inter professional so implementation that embraced that and used dyads of champions or leads for local implementation I think had more success”* (Canada-Ontario, Public, Participant 3).
Experience	*“… if they’re just newly qualified they’re, their knowledge, their skills, their leadership ability in terms of their pharmacy team might be very different to somebody who’s got 20 years of experience and more able to manage the team. And so that could be a major factor on, on whether an intervention is adopted or not”* (United Kingdom, Public, Participant 5).
**Context Attribute: Collaboration**	
Collaboration (generally)	*“We had 14 hospitals participating in [deidentified] that was the formal collaboration that did the evaluation, but mobilization has spread to more than 30 or 40 hospitals in [deidentified] through the provincial initiative senior friendly hospitals”* (Canada- Ontario, Public, Participant 3).
Networks	*“So, you know, in [deidentified] what we chose to do is we’ve got these strategic clinical networks and that’s great because they can, they can come up with the good ideas”* (Canada- Alberta, Private, Participant 1).
Partnerships	*“ Yeah I think it was, well we haven’t implemented the intervention as yet, but it definitely made it easier having partners in academic, or academia at the ready, like to be able to reach out to the university and be able to create a partnership. The partnership went very easily and we were I think that helped the project”* (Canada- Nova Scotia, Public, Participant 1).
Social interactions	*“To me the first thing that comes to my mind is the nature of the surroundings and the relationships among people in those surroundings in clinical setting”* (United States, Public, Participant 1).
**Context Attribute: Culture**	
Organizational culture	*“Context includes..... things like the culture of the organization as a whole, but also the cultures within the organization that maybe at a unit or program level and because they’re always different as well”* (Canada-Nova Scotia, Public, Participant 3).
**Context Attribute: Evaluation**	
Evaluation (generally)	*“So, we did the evaluation, that aspect of it was quantitative and what we showed from that was if the pharmacist was involved then you were more likely to get an evidence-based outcome. If more questions or information was exchanged you were more likely to get an evidence-based outcome and that sort of thing”* (United Kingdom, Public, Participant 5).
Routinized feedback	*“And sort the feedback loops that are occurring. So, the coal face is going about a change, they need to feed it up to a certain level, um, that are key leaders in saying, ‘Ok, I think, I think you need to keep going or we’re going to make a call and say, nope, we need to stop in this space.’ So that decision-making platform”* (Australia, Public, Participant 12).
Routinized audit	*“Yeah, and the, I think the hospital has, with handwashing, the hospital actually has a 90% benchmark like a target rate for handwashing so they audit that quite frequently and that quite good so what we observed was you know handwashing was done quite well and nurses have a lot of strategies to make sure they wash their hands longer than 30 seconds”* (Australia, Public, Participant 8).
Quality improvement monitoring	*“They have a lot of performance measures and probably in fact too many of them”* (United States, Public, Participant 3).
**Context Attribute: Facility Characteristics**	
Geography	*“Context, very heterogeneous and the public health needs of the populations vary very greatly around the different parts of the country… So there are very different needs in different parts of the country so that’s it’s a major driver of context”* (Canada- Alberta, Private, Participant 1).
Type of facility	*“ So in terms of the setting it would be like for example, you know, at the, at the kind of macro level what’s the healthcare setting, you know the type of model that you’re existing in. Then down at the hospital level is it an acute care hospital or long-term care hospital. Is it a university-affiliated or not”* (Canada- Nova Scotia, Public, Participant 5).
Size	*“We do know that larger hospitals have invested in internal change capacity and smaller hospitals or smaller provider organizations have struggled with that”* (Canada- Ontario, Public, Participant 1).
**Context Attribute: Financial Considerations**	
Funding	*“There is the challenge of the fact that they because this country has austerity budgets that there isn’t always funding in the local areas to deliver the kinds of programs that will make a difference to the public”* (United Kingdom, Public, Participant 2).
Costs	*“If one is talking about implementation, the primary obstacle to implementation certainly was cost. That was the #1 concern that any new intervention whether it was for quality or patient safety or affordability it had to be either cost neutral or it had to promise cost savings. And if you couldn’t do those things then the intervention really was a non-starter. So I think that cost was a primary barrier”* (United States, Public, Participant 1).
Funding model	*“It depends what kind of organization they’re working in. If they’re working for a state organization like the NHS most of it is, then there will be some organizational responsibility and they will also have a professional responsibility to do that. But we see more and more care being delivered through third sector organizations and they may perceive their responsibility if they perceive it at all, quite differently. If they’re independent sector organizations again it may be quite different depending if some of these organizations have doctors, nurses whoever working for them and they will, they’ll see some professional responsibility. But it may not be an organizational responsibility. And again if you look at these long-term I mean I have these organizations in my mind because I’ve been doing research with them recently, but these long-term residential care facilities they are typically privately owned and typically staffed by people with—and most of the care workers will not have a high level of clinical or even other education”* (United Kingdom, Public, Participant 4).
Financial incentives	*“So, I think a lot of it is context as well, is remuneration systems, there’s a lot of perverse incentives out there in the health system that encourages people, or certainly rewards people, to not do the best job they can”* (Australia, Public, Participant 3).
**Context Attribute: Governance**	
Governance (generally)	*“The kind of governance in organizations is really important”* (United Kingdom, Public, Participant 3).
Organizational structure	*“ Organisational structures that can actually support innovation in an organisation have to be very varied, well thought through…So in designing organisational structures that actually bring all of that together, rather than fragment, they’re exceedingly challenging. So I don’t for a minute suggest that to design a structure that would make all of that work is by any means easy but I will probably point out that I think one of our great impediments to innovation is actually being clear about which reporting line you should be working through to get change”* (Australia, Public, Participant 1).
Departmental approval	*“There’s another story with the challenges we’ve had, the bureaucrats and all those red tapes. They still haven’t passed after months we have been talking to them. It’s just a long process to get something approved [a digital documentation tool]*” (Australia, Public, Participant 8).
**Context Attribute: Leadership**	
Leadership (generally)	*“This top down system wide leadership worked was a really important part of the context in combination with this bottom up clinical leadership. And that system wide leadership was missing, so it was harder for them to make a decision whereby some organizations would lose out”* (United Kingdom, Public, Participant 3).
Mentorship	*“I that there are things people can do. There are actions that managers and leaders in organizations can take so that context is positively reinforcing or let’s say nudges people to behave in certain ways in the organization and not in other ways”* (United States, Public, Participant 1).
Organizational goals and priorities	*“When we’ve got our implementation priorities aligned with organizational priorities it’s easier…compared with if we’d gone along with something which was going to distract them from their key priorities then it would have been less welcome. And it would have been hard to have sold implemented interventions”* (United Kingdom, Public, Participant 6).
Champions	*“ So hands down you need to have local champions. You don’t have champions don’t even try and do it it’s a waste of time”* (Canada- Alberta, Private, Participant 1).
**Context Attribute: Organizational Readiness for Change**	
Buy-In	*“ That people, particularly practice nurses in paediatrics really need to feel that it is going to be a value add in what we’re doing in order to have buy-in and then for them to devote the energy and the resource that you’re gonna need in order to implement a change”* (Canada- Nova Scotia, Public, Participant 1).
Capacity	*“That people, particularly practice nurses in paediatrics really need to feel that it is going to be a value add in what we’re doing in order to have buy-in and then for them to devote the energy and the resource that you’re gonna need in order to implement a change ”* (Canada- Nova Scotia, Public, Participant 1).
Engagement	*“Like good ideas don’t go away and if clinicians are … heavily drawn into that process they will get what they want from their leaders”* (United States, Public, Participant 4).
**Context Attribute: Professional Role**	
Professional role (generally)	*“It’s not about valuing what one person does over another. It’s about understanding that #1 there’s more than enough work for everyone and how do we support the right person in the right place doing the right role for the patient? And, and within that have them understand each other’s roles that compliment and how they’re different”* (Canada- Nova Scotia, Public, Participant 2).
Clinical skill set	*“The other aspect is the knowledge; skills and understanding of the staff who could best use the evidence and put it into practice*” (United Kingdom, Public, Participant 2).
Professional education	*“Re healthcare workers…. How knowledgeable how educated are they on translation science? There’s individuals that are at the bedside so do you have a high percentage of very, you know, well-educated nurses? So they’re educated at the bachelors or graduate level or do we have predominantly associate level or practical nurses that are working. I think educational level makes a big difference in terms of the understanding of the use of evidence in making clinical practice changes and the implementation of those practice changes”* (United States, Public, Participant 2).
Accountability	*“You know, my professional group does this and we’re responsible to this and this is our accountability”* (Canada- Ontario, Public, Participant 3).
Job Autonomy	*“It’s their own decisions along capacity, time, resources, whatever decisional autonomy they have, whatever decisional autonomy the patient has”* (United Kingdom, Public, Participant 1).
Professional development	*“ Again so to them it’s well it’s their training; it’s their level of …, their level of CPD, Continuing Professional Development or other ongoing educational stuff”* (United Kingdom, Public, Participant 1).
Conflict	“*There was, you know, there was among the clinicians involved a certain amount of squabbling around about exactly which statin medication to put as the preferred medication in our formulary. So there was like, you know, some professional differences of opinion in people whose favourite drug was on it got more onboard. And people who didn’t, you know, who were not on the prevailing side of that argument were had a little bit of a not invented here situation but was someone else’s program because it wasn’t even the drug they wanted to use*” (United States, Public, Participant 3).
**Context Attribute: Resource Access**	
Resource access (generally)	*“You need resources at your disposal in order to collect it and this was the huge challenge across this organisation. There’s very little priority around equipping people with the resources to gather the evidence they need to make their case”* (United States, Public, Participant 3).
Lack of time	*“Our clinicians are so busy. … a primary care provider they have a panel of, you know, 2500 patients. They’re insanely busy they’re just not gonna have the time to be aware of these best practices or these guidelines or whatever”* (United States, Public, Participant 4).
Existence of guidelines	*“There’s a pretty constant attention to issues of quality and so issues of clinical guidelines and clinical implementation guidelines that is the creation of site-specific guidelines to figure out how to put into practice general clinical guidelines let’s say from national level quality consensus panels”* (United States, Public, Participant 1).
Formal communication	*“There’s a lot of communication networks that have already been existing in the system and one of those a newsletter that comes out every month. Another is a website that has the newsletter on that comes out every month”* (United States, Public, Participant 3)
Expert support	*“I was talking to some of my colleagues, saying, if only the hospitals had a position called KT consultants or something. It’s their role, they have to do it. Then it will sustain. Otherwise this is too difficult and especially with the knowledge as well. Sometimes they, you kind of have to kind of educate a lot of people along the way to do it, systematically so you don’t just implement something without a proper research process”* (Australia, Public, Participant 8).
Technology	*“Then, of course there are other things that are more easily identifiable like technology... Those things are certainly not unimportant. They certainly form an important part of the experience for individuals who are in, you know, modern work places”* (United States, Public, Participant 1).
Organizational training and education	*“You gonna publish this [guideline] … you have to have this educational process where you have to let people know about the pathway, bring them in, educate them about them, have them ask questions”* (Canada-Alberta, Private, Participant 1).
Documentation	*“And documentation was the main problem; there was, you know, a lot of research studies you know, documentation’s important in patient safety and all that but um, and it was 25% of people didn’t document anything anywhere. … we asked the people how they documented and tools they used…. So we designed a digital, ‘cause the hospital documentation system went digital probably a year before the project. But the surgical wound documentation was still in hardcopy”* (Canada-Ontario, Public, Participant 3).
Online Resources	*“This is online this is like your knowledge is consistently and always updated. It’s like Wikipedia it’s like when a new knowledge comes in boom it gets published and everybody sees it. Not like publishing these hard copy things, these PDFs that like old school. This is like everybody goes to this online portal that where the knowledge is kept up-to-date instantly and everybody can see it and knows that this is the latest stuff”* (Canada-Alberta, Public, Participant 4).
Equipment	*“And so one of the factors is the, the institution so even if a surgeon or wanted to do laparoscopic surgery if he or she worked at a hospital that didn’t have the laparoscopic equipment they wouldn’t do it”* (Canada-Ontario, Public, Participant 2).
Space as a resource	*“Sometimes they will have, maybe more likely to have, larger facilities. So they actually, even if they don’t have a private consulting room they will have a private room that they can take the patient to”* (Australia, Public, Participant 3).
**Context Attribute: System Features**	
Organizational support	*“Putting support systems in place that included daily huddles where you’re trying to start to do some of the implementation of the change. Getting, doing some job shadowing and data collection”* (Canada- Nova Scotia, Public, Participant 2).
Managing change	*“And then and so then it’s so those are some of the challenges of figuring out the logistics to stop those things happening. So, you know, how can you get the overhead pager turned off so it’s not, you know, it’s usually loud? How can you get pharmacies to, to change the automatic timing to that, you know, the statins aren’t given at 11:30 at night, for example?”* (Canada- Ontario, Public, Participant 5).
System complexity	*“I think a key challenge is related to the complexity or the under-estimation of the complexity of the system involved. … if you’ve done any work in a complex system, when you shift something in one place, something moves elsewhere that was unexpected”* (United Kingdom, Public, Participant 4).
Organizational stability	*“ So what’s happening within those regional organizations? They’ve got different dynamics going on. Some of them have new leadership and some of them have old leadership. Some of them have, I mean not old leadership, established leadership. [laugh] Some of them have different market dynamics and have a need to, different need to respond with, you know, different sort of standards and expectations of care in their local setting”* (United States, Public, Participant 3).
**Context Attribute: Work Structure**	
Work structure (generally)	*“If there’s a very authoritarian practice management structure, I think that tends to actually really influence the way people view change and generally I see implementation as change. If they see it as it being pushed down on them, they don’t give their ‘permission’ to actually undertake the work, and so it’s always, ‘I don’t have enough time.’ There’s a lot of reasons come out then, I think the management structure can have a major influence and whether or not people see themselves as working together, teamwork is embodied rather than just on paper”* (Australia, Public, Participant 4).
Teamwork	*“ So the work that we’ve done in surgery we’ve really gotten nurses, anaesthesiologists and surgeons to work together. And I think often various groups they’re not working together and so that’s a big barrier too they have to overcome”* (Canada- Ontario, Public, Participant 2).
Disconnect between research, education, and clinical care	*“It’s fantastic that we’ve all these scientists and amazing people who do research but how do we then get it back into practice? And I really feel that’s our missing link”* (Australia, Public, Participant 6).
Standardization of care	*“… what we’re aiming to do is standardize care across the province according to the best clinical practices and the pathway that the experts have identified approach across the organization”* (Canada- Ontario, Public, Participant 1).
Workload	*“The most difficult thing that we have to deal with is the overwhelming amount of other stuff we have to do. So it’s hard enough to do the day job without making time to do new stuff or even how you’d plan how you’re going to stop doing old stuff”* (United Kingdom, Public, Participant 6).
Workflow	*“I think a little more specifically it’s also the way that they currently accomplish work. The routines that they use the workflow patterns”* (United States, Public, Participant 1).
Scheduling	*“They had to totally rethink their staffing patterns including their core hours they worked and when they all took, when everybody on the team took their vacations because they no longer had a predictable pattern of when people were coming for appointments”* (United Kingdom, Public, Participant 4).
Continuity of care	*“We looked at community pharmacists’ screening for absolute risk of cardiovascular disease. The main challenge, that was the sort of clinical issue, the main challenge was the continuity of care between pharmacies and general practice”* (Australia, Public, Participant 3).
**Context Attribute: Political Climate**	
Political climate (generally)	*“ So the broader political climate it’s a big piece which is, you know, close after the government of the day and elected *representatives” (United Kingdom, Public, Participant 1).
**Context Attribute: Regulatory and Legislative Standards**	
Regulatory and legislative standards (generally)	*“There’s a wider context of say the regulatory framework or whatever that people are operating in ‘ cause they’ll be different, there can be different policies depending on the jurisdiction that you’re in”* (Australia, Public, Participant 2).
Provincial responsibility	*“There’s a provincial role or more of a provincial role we should take in supporting clinical networks if, if what we want is to support, you know, standardizing practice and supporting best practice. So like in some areas we have, we have put I would say tremendous provincial resource into supporting clinical networks and the cancer system’s a good example”* (Canada-Ontario, Public, Participant 3).
Standard of practice or care	*“I would also say that at the time that standards or indicators or, you know, even innovative practices that we’re trying to promote [laugh] at this time that they’re being identified having a mix of perspectives that bring those that context to that discussion is also important”* (Canada-Ontario, Public, Participant 4).
Accreditation	*“ So accreditation, we work with two accrediting bodies, one is Accreditation Canada and the other one is the College of Physicians.... So Accreditation Canada does largely, you know, our system accreditation and the College of Physicians does our lab and some smaller clinic kinds of accreditation for us at this point in time”* (Canada-Alberta, Public, Participant 4).
Legal	*“The way we’re structured, the local medical groups are really completely separate entities. They’re not just different divisions of the same body, they’re legally structured differently”* (United States, Public, Participant 3).

Abbreviation: KT, knowledge translation.

**Table 4:** Presence of Context Attributes (n = 16) and Their Features (n = 66) by Country (n = 4)

**Attribute**	**Feature**	**Country**			
		**Australia**	**Canada**	**UK**	**USA**
Patient characteristics	Patient demographics	√	√	√	√
	Patient expectations and preferences	√	√	√	√
Health professional characteristics	Group composition	√	√	√	√
	Experience	√	√	√	√
Collaboration	Collaboration (generally)	√	√	√	√
	Networks	√	√	√	-
	**Partnerships**	√	√	-	-
	**Social interactions**	-	-	-	√
Culture	Organizational culture		√	√	√
Evaluation	Evaluation (generally)	√	√	√	√
	Routinized feedback		√	-	√
	Routinized audit	√	√	-	√
	**Quality improvement monitoring**	-	√	-	√
Facility characteristics	Geography	√	√	√	√
	Type of facility	√	√	√	-
	**Size**	√	√	-	-
Financial considerations	Funding	√	√	√	√
	Costs	√	√	√	√
	Funding model	√	-	√	√
	Financial incentives	√	√	√	√
Governance	Governance (generally)	√	√	√	√
	Organizational structure	√	√	√	√
	**Departmental approval**	√	-	-	-
Leadership	Leadership (generally)	√	√	√	√
	Organizational goals and priorities	√	√	√	√
	Champions	√	√	√	√
	**Mentorship**	-	√	-	-
Organizational readiness for change	Buy-In	√	√	√	√
	Capacity	√	√	√	√
	Engagement	√	√	√	√
Professional role	Professional role (generally)	√	√	-	√
	Clinical skill set	√	√	√	√
	Professional role training	√	√	√	√
	Accountability	√	√	-	√
	Job autonomy	-	√	√	√
	Professional development	-	√	√	√
	Conflict	√	√	-	√
Resource access	Resource access (generally)	√	√	√	√
	Lack of time	√	√	√	√
	Existence of guidelines	√	√	√	√
	Formal communication	√	√	-	√
	Expert support	√	√	-	√
	Technology	√	√	-	√
	Organizational training and education	√	√	√	√
	**Documentation**	√	√	-	-
	Online resources	√	√	-	√
	**Equipment**	√	√	-	-
	**Space as a resource**	√	√	-	-
System Features	Organizational support	√	√	√	√
	Managing change	√	√	-	√
	**System complexity**	-	√	√	-
	Organizational changes	√	√	√	√
Work structure	Work structure (generally)	√	√	√	√
	Teamwork	√	√	-	√
	Disconnect between research, education, and clinical care	√	√	-	√
	Standardization of care	√	√	√	√
	Workload	√	√	√	-
	Workflow	√	√	-	√
	**Scheduling**	√	-	√	-
	**Continuity of care**	√	-	-	√
Political climate	Political climate (generally)	√	√	√	√
Regulatory and legislative standards	Regulatory and legislative standards (generally)	√	√	-	√
	**Provincial responsibility **	-	√	-	-
	Standard of practice or care	√	√	√	√
	Accreditation	√	√	-	√
	**Legal**	√	-	-	√

Ö = mentioned by at least 1 interviewee in the designated country. - = not mentioned by any interviewees in the designated country.

**Table 5:** Context Attributes (n = 16) and Features (n = 66) by KT Experience and Primary Role

**Attribute**	**Feature**	**Experience**		**Primary Role**	
		**Less (0-5 Years)** **(n = 11)**	**More (>5 Years)** **(n = 28)**	**Researcher** **(n = 20)**	**Change Agent** **(n = 19)**
Patient characteristics	Patient demographics	√	√	√	√
	Patient expectations and preferences	√	√	√	√
Health professional characteristics	Group composition	√	√	√	√
	Experience	√	√	√	√
Collaboration	Collaboration (generally)	√	√	√	√
	Networks	√	√	√	√
	Partnerships	√	√	√	√
	Social interactions	√	√	√	-
Culture	Organizational culture	√	√	√	√
Evaluation	Evaluation (generally)	√	√	√	√
	Routinized feedback	√	√	√	√
	Routinized audit	√	√	√	√
	Quality improvement monitoring	-	√	√	√
Facility characteristics	Geography	√	√	√	√
	Type of facility	√	√	√	√
	Size	√	√	√	√
Financial considerations	Funding	√	√	√	√
	Costs	√	√	√	√
	Funding model	√	√	√	√
	Financial incentives	-	√	√	√
Governance	Governance (generally)	√	√	√	√
	Organizational structure	√	√	√	√
	Departmental approval	√	√	√	-
Leadership	Leadership (generally)	√	√	√	√
	Organizational goals and priorities	√	√	√	√
	Champions	√	√	√	√
	Mentorship	-	√	√	√
Organizational readiness for change	Buy-In	√	√	√	√
	Capacity	√	√	√	√
	Engagement	√	√	√	√
Professional role	Professional role (generally)	√	√	√	√
	Clinical skill set	√	√	√	√
	Professional role training	√	√	√	√
	Accountability	√	√	√	√
	Job autonomy	√	√	√	√
	Professional development	-	√	√	√
	Conflict	√	√	√	√
Resource access	Resource access (generally)	√	√	√	√
	Lack of time	√	√	√	√
	Existence of guidelines	√	√	√	√
	Formal communication	√	√	√	√
	Expert support	√	√	√	√
	Technology	√	√	√	√
	Organizational training and education	√	√	√	√
	Documentation	√	√	√	√
	Online resources	√	√	√	√
	Equipment	-	√	√	√
	Space as a resource	-	√	√	√
System features	Organizational support	√	√	√	√
	Managing change	√	√	√	√
	System complexity	√	√	√	√
	Organizational stability	√	√	√	√
Work structure	Work structure (generally)	√	√	√	√
	Teamwork	√	√	√	√
	Disconnect research, education, care	√	√	√	√
	Standardization of care	√	√	√	√
	Workload	-	√	√	√
	Workflow	√	√	√	-
	Scheduling	√	√	√	√
	Continuity of care	-	√	√	-
Political climate	Political climate (generally)	√	√	√	√
Regulatory and legislative standards	Regulatory and legislative standards (generally)	√	√	√	√
	Provincial responsibility	-	√	-	√
	Standard of practice or care	√	√	√	√
	Accreditation	√	√	√	√
	Legal	√	√	√	√

Abbreviation: KT, knowledge translation. √ = mentioned by at least 1 interviewee in the designated category. - = not mentioned by any interviewees in the designated category.

 Two of the 16 context attributes were identified in all 39 interviews: (1) *Culture, *defined as ‘the inherited beliefs, values, and attitudes of a group’ and (2) *Resource Access*, defined as ‘access to resources of any kind.’ *Culture* contained a single feature – organizational culture. An illustrative quote for this feature is:

*“Context includes … things like the culture of the organization as a whole, but also the cultures within the organization that maybe at a unit or program level and because they’re always different as well” *(Canada, Participant 3).

*Resource Access* contained 11 features: (1) resource access (generally), (2) lack of time, (3) existence of guidelines, (4) formal communication, (5) expert support, (6) technology, (7) organizational training and education, (8) documentation, (9) online resources, (10) equipment, and (11) space as a resource. An illustrative quote for resource access (generally) is:

*“You need resources at your disposal in order to collect it and this was the huge challenge across this organisation. There’s very little priority around equipping people with the resources to gather the evidence they need to make their case” *(United States, Participant 3).

####  Context Attributes and Features by Country

 Of particular interest in this study was whether and which attributes and features of context varied by country. Table 4 indicates the presence or not of the 16 attributes of context and their features by country. All 16 attributes were discussed by key informants in each country. Most features (n = 52 of 66) were also discussed by key informants from at least three of the four countries. Only 3 of the 66 features were mentioned by key informants in a single country, with no discernable pattern. Two features were mentioned by only Canadian key informants: (1) mentorship (attribute*: Leadership*) and (2) provincial responsibility (attribute: *Regulatory and Legislative Standards*). A third feature – departmental approval (attribute: *Governance*) was discussed only by Australia key informants. Illustrative quotes for these features can be found in Table 3. There were also differences by country in terms of some features being less frequently mentioned by key informants of a particular country. For example, of the 66 total identified features of context identified, 26 of them were not reported by UK key informants compared to only 12, 7, and 6 features not being identified by the United States, Australian, and Canadian key informants, respectively. A summary of identified features by country can be seen in Table 4.

####  Context Attributes and Features by Level of Experience 

 As described earlier, key informants were categorized based on whether they had ‘less’ or ‘more’ experience in KT using 5 years as the cut point; key informants with 5 years or less of KT experience were categorized as ‘less experienced’ and those with more than 5 years of experience were categorized as ‘more experienced.’ Table 5 displays the presence or not of all 16 attributes and 66 features of context by level of KT experience (less or more) of the key informants. There was considerable consistency with respect to context attributes and features identified by key informants with less and more experience in KT. All 16 attributes and the vast majority of features (57 of 66) were discussed by both more and less experienced key informants. Only 9 features were present in one group and not the other, all of which were mentioned by stakeholders with more experience and not those with less experience. The 9 features were: quality improvement monitoring (attribute: *Evaluation*), financial incentives (attribute: *Financial Considerations*), mentorship (attribute: *Leadership*), professional development (attribute: *Professional Role*), equipment (attribute: *Resource Access*), space as a resource (attribute: *Resource Access*), workload (attribute: *Work Structure*), continuity of care (attribute: *Work Structure*), and provincial responsibility (attribute: *Regulatory *&* Legislative Standards*).

####  Context Attributes and Features by Primary Role 

 While all key informants in this study were health system stakeholders responsible for the design and implementation of interventions, programs, and change processes, they were asked to self-identify their primary role as either a change agent/KT specialist or KT researcher. Table 5, in addition to level of experience, also displays the presence or not of all 16 attributes and 66 features of context by primary role of the key informants. Similar to country and level of KT experience, all context attributes and most features (n = 61 of 66) were mentioned by key informants in both primary roles. Four features were discussed by KT researchers but not change agents/KT specialists: (1) workflow (attribute*: Work Structure*), (2) continuity of care (attribute*: Work Structure*), (3) social interactions (attribute*: Collaboration*), and (4) departmental approval (attribute*: Governance*). One feature was discussed by change agents/KT specialists but not KT researchers: provincial responsibility (attribute: *Regulatory and Legislative Standards*).

## Discussion

###  Summary of Findings

 The purpose of this study was to compile a list of contextual attributes and their features relevant to KT in health settings from the perspective of health system stakeholders (change agents/KT specialists and KT researchers). We codified tacit knowledge of these stakeholders in four countries about what constitutes context. In total, we identified 66 unique features of context, categorized into 16 broader attributes. The 16 attributes of context covered all contextual issues identified in all 39 interviews. We found instances of all 16 attributes of context in the interviews irrespective of country, level of experience with KT, and primary role (change agent/KT specialist vs. KT researcher), revealing robustness and transferability of our attributes of context. Some variation in context was identified at the finer-grained feature level (of these attributes) but even then, most features were relevant across countries, KT experience levels and primary role.

###  New Attributes of Context Identified and their Features 

 We compared the 66 features of context identified in this study to those contained in the TICD framework checklist,^60^ a recent comprehensive published consolidation of KT checklists based on frameworks which include context. The TICD includes elements that are in popular meta-typologies such as the CFIR,^44^ as well as commonly used conceptual frameworks such as the Theoretical Domains Framework^42,43^ and the PARiHS Framework^48^. Almost all of our context attributes (n = 15 of 16) mapped to concepts in the TICD, supporting international consensus for these attributes. The one attribute from our analyses that is not represented in TICD is ‘Facility Characteristics.’ Three features were identified in our study that characterize this attribute: geography, type of facility, and size.

 While many of our 16 higher-level attributes of context were known previously, they were not well-defined. Through this study, we synthesized experiential knowledge about context attributes and provide a large range of illustrative features for them. For instance, almost half of the features (n = 27 of 66) identified by key informants in this study are not contained in the TICD checklist. This includes new features in most (n = 12 of 16) attributes. We identified new features of several commonly discussed context attributes: (1) attribute* Resource Access *(new features – existence of guidelines, technology, documentation, online resources); (2) attribute* Leadership *(new feature – mentorship); (3) attribute* Work Structure *(new features – work structure generally, disconnect between research, education, and clinical care, standardization of care, workload, scheduling, continuity of care); (4) *Attribute Financial *(new feature – funding model); (5) attribute* Collaboration *(new features –partnerships, social interactions); (6) *Attribute Professional Role *(new feature – accountability, job autonomy); and attribute* System Features *(new features –managing change, system complexity, organizational stability). We also identified new features for some less commonly discussed context attributes including: (1) attribute* Professional Role *(new features – accountability, job autonomy); (2) attribute* Evaluation *(new feature – routinized audit); (3) attribute* Patient Characteristics *(new feature – patient demographics); (4) attribute* Governance *(new feature – departmental approval); and (5) attribute* Regulatory and Legislative Standards *(new features – provincial responsibility, accreditation).

 One reason why many of our features might be lacking from the TICD and other meta-typologies such as the CFIR may be because that these frameworks focus mainly on higher level context attributes rather than the more detailed context features. Our identification of these features is a critical advancement in the KT field. As a result of this work, we advance much needed conceptual clarity in context, which is critical to develop common assessment tools to measure context to determine which specific context features are more or less important in different contexts and for changing different healthcare professional behaviours. The expectation is that such measurement tools could subsequently be used to: (1) tailor KT intervention designs and their delivery, (2) better interpret the effects of KT interventions, and (3) pragmatically guide change agents and researchers in their KT efforts.

###  Common Attributes of Context

 Next we discuss the more commonly identified context attributes that surfaced in our interviews with stakeholders internationally.

####  Culture

 All 39 key informants in this study identified the context attribute *Culture* as relevant to KT. This finding is consistent with previous studies examining stakeholder perspectives on facilitators of and barriers to KT,^74-80^ suggesting broad agreement among stakeholders internationally of the importance of culture. This is important because consensus among organizational leaders on the factors important for KT has been associated with greater success in the implementation of evidence-based practices.^81^

 The feature ‘organizational culture,’ which refers to the normative beliefs and shared expectations that govern the work behaviour of a clinical team or employees of a healthcare facility, was mentioned by key informants across all four countries in this study. This is consistent with past research. For example, Beidas and colleagues,^74^ in a recent study of stakeholders’ perspectives of the facilitators of and barriers to implementing evidence-based practices in a United States public mental health system, found agencies that were successful in implementation were those that “changed their culture to fit the needs of the evidence-based practice” (p. 897). Similarly, Harvey and colleagues,^75^ who investigated which contextual factors are most influential in mediating performance improvements in the United Kingdom (England and Scotland) public health systems, found that organizations with successful implementation of evidence-based practices were those that had organizational leadership and management that promoted a “can do” culture.

####  Resource Access 

 The attribute *Resource Access* was also identified by all key informants across all countries in our study. The importance of *Resource Access* was acknowledged in multiple previous studies.^74,75,77-80,82,83^ Specifically, previous studies with health system stakeholders found that lack of appropriate resources such as insufficient staffing,^78-80,83^ lack of appropriate educational and training programs,^74,75,78,83^ and insufficient support and poor communication^75,83^ hindered implementation efforts. In the present study, the most commonly discussed *Resource Access *feature was lack of time. This is consistent with previous research indicating the need for protected time for KT efforts (eg, supervision, training and education) to ensure consistent and maintained engagement by staff and leaders.^74,79,82,83^ The importance of lack of time may also reflect individuals’ unwillingness to partake in uncompensated work tasks or activities that take time away from more pressing patient care demands. This feature may be particularly important for individuals who must balance many competing demands on their time.

 While the attribute *Resource Access* was mentioned by all key informants on our study, it is unclear why certain features of the attribute were viewed as more important (ie, mentioned by more key informants) than other features within the attribute. For example, the feature of lack of time was discussed by the majority of key informants in all four countries, while the features equipment, and space as a resource were both only mentioned by one key informant in each of Australia and Canada. This may reflect that having enough time to implement an innovation is crucial regardless of what the innovation is. Likewise, having sufficient equipment and space may be specific to particular innovations and thus not a universal important component of context necessary for successful KT. However, it may also be that participants in our study had sufficient experience in KT that they did not require special equipment or space and therefore did not view those components as important contextual features.

####  Organizational Readiness for Change

 All three features of *Organizational Readiness for Change *were commonly mentioned by the key informants in all four countries in our study: (1) buy-in (agreeing with and accepting a suggestion or change (eg, a new policy)); (2) capacity (the organization’s total workload for running current operations and could include conducting change activities); and (3) engagement (the meaningful involvement of staff or stakeholders in the delivery of healthcare services). The identification of these features as important to KT are also consistent with previous reports of stakeholder perspectives.^74,75,80^ Beidas and colleagues^74^ found that agencies with early and sustained engagement experienced successful implementation of evidence-based practices more often than did agencies without capacity and engagement. Similarly, lack of buy-in is suggested to impede KT because individuals who are not fully committed to the implementation are thought to be less motivated to contribute to and ensure the success of the project.^74,75,80^

####  Collaboration, Financial Considerations, Leadership, Work Structure

 Other more common attributes identified in our study included: *Collaboration, Financial Considerations, Leadership*, and *Work Structure*. Previous studies investigating stakeholders’ perspectives of factors important for KT have also identified leadership as a core feature. Specifically, the presence of committed and supportive leaders and managers who are responsive to KT has been noted as important.^75,76,82,83^ Similarly, components of *Work Structure,*^74,80^*Financial Considerations,*^76,77,79,80^ and *Collaboration* between stakeholders^74-76,78-80^ have also been identified as key features of context important to KT by diverse stakeholders internationally. With respect to *Work Structure*, Beidas and colleagues^74^ in a study of stakeholders from the United States found that teamwork (a feature of our context attribute *Work Structure*) was important to KT in that it facilitates buy-in (a feature of our context attribute *Organizational Readiness for Change*) and setting organizational goals and priorities (a feature of our context attribute *Leadership*). Financial features identified in previous studies from several countries were similar to those we identified. Financial features were found to facilitate KT when perceived cost was low or known,^79,80^ funding was available for the implementation,^79^ and incentives were offered to encourage buy-in towards the implementation^76^; and were found to hinder KT when cost was high or unknown.^77^ Internationally, multiple studies with stakeholders have also identified the importance of collaboration between stakeholders, both internal and external to the organization.^74-76,78-80^ Beidas and colleagues^74^ in their study of United States stakeholders found that coordinated collaboration across stakeholder groups throughout the whole KT process was important to achieve successful implementation of evidence-based practices. Muellmann and colleagues,^78^ in a study of stakeholders across five countries (Belgium, Germany, Ireland, Norway, and Poland) found that in addition to collaboration during KT, continued collaboration and communication between stakeholders (after implementation) was also necessary to facilitate successful KT. Similar to our study, Harvey et al,^75^ Schneider and colleagues,^80^ and Renz and colleagues^79^ in studies with stakeholders in the United Kingdom (England and Scotland) and United States, found specifically that extending collaboration to external organizations/stakeholders was important to successful KT.

###  Less Commonly Reported Features of Context

 While the 16 attributes of context identified in this study varied very little by country, stakeholder level of experience with KT (less/more experienced) and stakeholder primary role (change agent/KT specialist vs. KT researcher), there was some (although limited) variation as expected in the finer grained features of context. While all features were mentioned by multiple stakeholders, 14 of the 66 features were reported less consistently (ie, in fewer than 3 of the 4 countries in our study). The majority of these 14 features were reported by key informants regardless of their level of experience with KT or their primary role. Furthermore, 11 of them were identified by stakeholders in more than 1 country. The only notable trend was that most of the uncommon features (n = 12 of 14) were not identified by stakeholders in the United Kingdom. However, stakeholders from United Kingdom in general, in this study, spontaneously identified fewer features of context (n = 40 features) compared to the United States (n = 54), Australia (n = 59) and Canada (n = 60). We do not know from this study or the literature why some features of context would be identified less frequently overall or by stakeholders in the United Kingdom in particular, or if these less frequent features are less important to KT. Since specific features of context were not specifically probed in our study, it is possible that more stakeholders would have identified the less commonly reported features had they been deliberately asked about them. Therefore, at this stage of our research program, we have retained all context features identified on our list of features in order to not underestimate the importance of any of the features. This study comprised a necessary first step of codifying tacit knowledge of stakeholders about the nature of context, thereby advancing our understanding of context in KT.

###  Next Steps

 In this study we identified context attributes and features perceived by health system stakeholders in four developed countries to be relevant to KT in health settings. We suggest that a similar study to the one reported here be conducted with stakeholders in developing countries to determine if the same or different features of context apply in developing countries. Simultaneously to the study reported in this manuscript, we also identified attributes and features of context by researchers^59^ and healthcare professionals.^61^ Our next steps include triangulation across all three studies to produce a unified framework of context for implementation that can be useful to health system stakeholders – both change agents/KT specialists and KT researchers. Our goal is for change agents/KT specialists to be able to use the framework to: pragmatically guide their implementation efforts by identifying the important features of context to consider when choosing, designing and implementing interventions; and to help assess the transferability of successful interventions from other contexts to their own (by identifying contextual features they need to have in place for successful KT).^84^ KT Researchers will be able to use the framework to guide *a priori *assessments of context (to assist in the design and delivery of their interventions) as well as *posteriori* assessments of context (to aid in their interpretation of the effects of interventions which can then inform the design and delivery of their subsequent trials).

 We acknowledge that the direction and extent of influence of all context features depend on the extent to which the particular feature is lacking or sufficiently available (eg, time) and the degree of importance of that particular feature to a specific KT initiative/strategy. In this study, we did not set out to establish whether a feature has a positive or negative effect and under what conditions; instead our purpose was to develop a comprehensive list of features of context. Future research should focus on trying to elucidate under what conditions the context features from this study positively or negatively empirically influence KT. A realist review approach might be well-suited to investigating under what conditions a feature has a specific effect in what kind of KT strategy.

###  Limitations and Strengths 

 Our main limitation is that we asked stakeholders about their perceptions about what contextual factors influence KT to surface their experiential (tacit) evidence on this topic. We do not know at this stage if the identified factors are important moderators of KT strategies or if they have a positive or negative effect on KT and under what conditions. Second, we did not ask stakeholders about specific context features, therefore we cannot state that selected features, if not mentioned by stakeholders in a specific country, are not important in their country, only that they did not spontaneously mention them as important context features. Third, we only interviewed stakeholders from clinical practice settings (acute care, primary care); stakeholders from other health settings (eg, community, public health) may provide differing views. Fourth, while each of the countries reflected in our sample have dominantly a publicly funded system, each also has a private component to their healthcare system. However, our sample largely reflects stakeholders from the public systems (n = 33 of 39). Fifth, despite conducting a relatively large number of interviews, our sample was limited to stakeholders in four high-income countries. Incorporating the views from stakeholders from low- and middle-income countries may provide a potentially more complete or nuanced picture of the attributes and features of context important for KT success internationally. Given these limitations, the attributes and features of context presented in this paper should be considered provisional until further research is done to confirm these findings in other settings.

 The main strength of this study is our sample, which included stakeholders from multiple countries and different healthcare systems. This allowed us to identify attributes and features of context relevant to a broader range of stakeholders and settings than in previous studies.

## Conclusion

 Through this study, we identified 66 unique features of context, grouped into 16 broader attributes of context, perceived as relevant to KT in health settings by a variety of health system stakeholders internationally. There was considerable consistency in the 16 attributes and 66 features identified irrespective of the stakeholder’s country, amount of KT experience, and primary role (change agent/KT specialist or KT researcher), suggesting transferability of the attributes and features of context identified in this study. This is the largest study to date that we are aware of with health system stakeholders that has resulted in the identification of a large number of attributes and their features of context perceived to be relevant to KT. Further work is needed to confirm these findings across a broader range of countries, including low and middle-income countries.

## Ethical issues

 This study was approved by the Ottawa Health Science Network Research Ethics Board (affiliated with the University of Ottawa, Ottawa, ON, Canada) and the Deakin University Health Ethics Advisory Group (affiliated with Deakin University, Burwood, VIC, Australia). Ethics approval was only sought in Canada and Australia as this is where the interviews were conducted and data stored and analyzed. Ethics approval was not sought from the United States or the United Kingdom as participants from these countries were contacted by phone by research team members in Canada. All participants provided written informed consent.

## Competing interests

 Authors declare that they have no competing interests.

## Authors’ contributions

 All authors (JES, AMH, MC, KB, JC, JMG, KD, LA, JB, JJF, NI, JL, SM, MH, TN, JV, and IDG) participated in conception of the study. JES, AMH, JC, JMG, JB, JJF, NI, JL, SM, MH, TN, JV, and IDG participated in securing funding for the project. All authors (JES, AMH, MC, KB, JC, JMG, KD, LA, JB, JJF, NI, JL, SM, MH, TN, JV, and IDG) participated in designed the data analysis plan. KB, KD, and AH conducted the interviews. KB and KD led the coding of the data. JES, IDG, KB, KD, LA, MC, and AMH participated in the analysis of the coded data and all authors (JES, AMH, MC, KB, JC, JMG, KD, LA, JB, JJF, NI, JL, SM, MH, TN, JV, and IDG) provided critical input into the analysed data. JES, AMH, KB, MC, and LA drafted the manuscript. All authors (JES, AMH, MC, KB, JC, JMG, KD, LA, JB, JJF, NI, JL, SM, MH, TN, JV, and IDG) provided input and critical feedback on the manuscript and approved the final manuscript. Consent for publication was obtained from all co-authors (JES, AMH, MC, KB, JC, JMG, KD, LA, JB, JJF, NI, JL, SM, MH, TN, JV, and IDG).

## References

[1] Lang ES, Wyer PC, Haynes RB (2007). Knowledge translation: closing the evidence-to-practice gap. Ann Emerg Med.

[2] Lauer MS, Skarlatos S (2010). Translational research for cardiovascular diseases at the National Heart, Lung, and Blood Institute: moving from bench to bedside and from bedside to community. Circulation.

[3] McGlynn EA, Asch SM, Adams J (2003). The quality of health care delivered to adults in the United States. N Engl J Med.

[4] Schuster MA, McGlynn EA, Brook RH (1998). How good is the quality of health care in the United States?. Milbank Q.

[5] Grol R (2001). Successes and failures in the implementation of evidence-based guidelines for clinical practice. Med Care.

[6] Villar J, Carroli G, Gülmezoglu AM (2001). The gap between evidence and practice in maternal healthcare. Int J Gynaecol Obstet.

[7] Mellis C (2015). Evidence-based medicine: what has happened in the past 50 years?. J Paediatr Child Health.

[8] Straus SE, McAlister FA (2000). Evidence-based medicine: a commentary on common criticisms. CMAJ.

[9] Implementation Science. About. 2020. https://implementationscience.biomedcentral.com/about.

[10] Nilsen P (2015). Making sense of implementation theories, models and frameworks. Implement Sci.

[11] Greenhalgh T, Robert G, Macfarlane F, Bate P, Kyriakidou O (2004). Diffusion of innovations in service organizations: systematic review and recommendations. Milbank Q.

[12] Backer TE (1991). Knowledge utilization: the third wave. Knowledge.

[13] Landry R, Amara N, Lamari M (2001). Climbing the ladder of research utilization: evidence from social science research. Sci Commun.

[14] Rich RF, Oh CH (1994). The utilization of policy research.

[15] Dogherty EJ, Harrison MB, Graham ID (2010). Facilitation as a role and process in achieving evidence-based practice in nursing: a focused review of concept and meaning. Worldviews Evid Based Nurs.

[16] Kimberly J, Cook JM (2008). Organizational measurement and the implementation of innovations in mental health services. Adm Policy Ment Health.

[17] Mitton C, Adair CE, McKenzie E, Patten SB, Waye Perry B (2007). Knowledge transfer and exchange: review and synthesis of the literature. Milbank Q.

[18] Squires JE, Estabrooks CA, O’Rourke HM, Gustavsson P, Newburn-Cook CV, Wallin L (2011). A systematic review of the psychometric properties of self-report research utilization measures used in healthcare. Implement Sci.

[19] Squires JE, Estabrooks CA, Gustavsson P, Wallin L (2011). Individual determinants of research utilization by nurses: a systematic review update. Implement Sci.

[20] Squires JE, Hutchinson AM, Boström AM, O’Rourke HM, Cobban SJ, Estabrooks CA (2011). To what extent do nurses use research in clinical practice? a systematic review. Implement Sci.

[21] Glaser EM, Abelson HH, Garrison K. Putting Knowledge to Use: Facilitating the Diffusion of Knowledge and the Implementation of Planned Change. San Francisco: Jossey-Bass; 1983.

[22] Lewis CC, Fischer S, Weiner BJ, Stanick C, Kim M, Martinez RG (2015). Outcomes for implementation science: an enhanced systematic review of instruments using evidence-based rating criteria. Implement Sci.

[23] Chaudoir SR, Dugan AG, Barr CH (2013). Measuring factors affecting implementation of health innovations: a systematic review of structural, organizational, provider, patient, and innovation level measures. Implement Sci.

[24] LaRocca R, Yost J, Dobbins M, Ciliska D, Butt M (2012). The effectiveness of knowledge translation strategies used in public health: a systematic review. BMC Public Health.

[25] Reichenpfader U, Carlfjord S, Nilsen P (2015). Leadership in evidence-based practice: a systematic review. Leadersh Health Serv (Bradf Engl).

[26] Wozney L, McGrath PJ, Gehring ND (2018). eMental healthcare technologies for anxiety and depression in childhood and adolescence: systematic review of studies reporting implementation outcomes. JMIR Ment Health.

[27] Li SA, Jeffs L, Barwick M, Stevens B (2018). Organizational contextual features that influence the implementation of evidence-based practices across healthcare settings: a systematic integrative review. Syst Rev.

[28] De Vries H, Bekkers V, Tummers L (2016). Innovation in the public sector: a systematic review and future research agenda. Public Adm.

[29] Kaplan HC, Brady PW, Dritz MC (2010). The influence of context on quality improvement success in health care: a systematic review of the literature. Milbank Q.

[30] Lau R, Stevenson F, Ong BN (2016). Achieving change in primary care--causes of the evidence to practice gap: systematic reviews of reviews. Implement Sci.

[31] Ovretveit J. Change Achievement Success Indicators (CASI). Stockholm, Sweden: Karolinska Institute Medical Management; 2004.

[32] Rycroft-Malone J (2004). The PARIHS framework--a framework for guiding the implementation of evidence-based practice. J Nurs Care Qual.

[33] May C, Finch T, Mair F (2007). Understanding the implementation of complex interventions in health care: the normalization process model. BMC Health Serv Res.

[34] Berta W, Ginsburg L, Gilbart E, Lemieux-Charles L, Davis D (2013). What, why, and how care protocols are implemented in Ontario nursing homes. Can J Aging.

[35] Fleuren MA, Paulussen TG, Van Dommelen P, Van Buuren S (2014). Towards a measurement instrument for determinants of innovations. Int J Qual Health Care.

[36] McDonald KM, Schultz EM, Chang C (2013). Evaluating the state of quality-improvement science through evidence synthesis: insights from the closing the quality gap series. Perm J.

[37] Dixon-Woods M. The Problem of Context in Quality Improvement. London: The Health Foundation; 2014.

[38] Estabrooks CA (2003). Translating research into practice: implications for organizations and administrators. Can J Nurs Res.

[39] Hutchinson AM, Mallidou AA, Toth F, Cummings GG, Schalm C, Estabrooks CA. Review and Synthesis of Literature Examining Characteristics of Organizational Context that Influence Knowledge Translation in Healthcare: Technical Report. Edmonton, Alberta: University of Alberta; 2010.

[40] Meijers JM, Janssen MA, Cummings GG, Wallin L, Estabrooks CA, R YGH (2006). Assessing the relationships between contextual factors and research utilization in nursing: systematic literature review. J Adv Nurs.

[41] French B (2005). Contextual factors influencing research use in nursing. Worldviews Evid Based Nurs.

[42] Cane J, O’Connor D, Michie S (2012). Validation of the theoretical domains framework for use in behaviour change and implementation research. Implement Sci.

[43] Michie S, Johnston M, Abraham C, Lawton R, Parker D, Walker A (2005). Making psychological theory useful for implementing evidence based practice: a consensus approach. Qual Saf Health Care.

[44] Damschroder LJ, Aron DC, Keith RE, Kirsh SR, Alexander JA, Lowery JC (2009). Fostering implementation of health services research findings into practice: a consolidated framework for advancing implementation science. Implement Sci.

[45] Champagne F. Discussion Paper No.39: The Ability to Manage Change in Health Care Organizations. Commission on the Future of Health Care in Canada; 2002.

[46] Graham ID, Logan J, Harrison MB (2006). Lost in knowledge translation: time for a map?. J Contin Educ Health Prof.

[47] McNulty T, Ferlie E. Reengineering Health Care: The Complexities of Organizational Transformation. New York: Oxford University Press; 2002.

[48] Kitson A, Harvey G, McCormack B (1998). Enabling the implementation of evidence based practice: a conceptual framework. Qual Health Care.

[49] Kitson AL, Rycroft-Malone J, Harvey G, McCormack B, Seers K, Titchen A (2008). Evaluating the successful implementation of evidence into practice using the PARiHS framework: theoretical and practical challenges. Implement Sci.

[50] Flottorp SA, Oxman AD, Krause J (2013). A checklist for identifying determinants of practice: a systematic review and synthesis of frameworks and taxonomies of factors that prevent or enable improvements in healthcare professional practice. Implement Sci.

[51] Brennan SE, Bosch M, Buchan H, Green SE (2012). Measuring organizational and individual factors thought to influence the success of quality improvement in primary care: a systematic review of instruments. Implement Sci.

[52] Estabrooks CA, Squires JE, Cummings GG, Birdsell JM, Norton PG (2009). Development and assessment of the Alberta Context Tool. BMC Health Serv Res.

[53] Helfrich CD, Li YF, Sharp ND, Sales AE (2009). Organizational readiness to change assessment (ORCA): development of an instrument based on the Promoting Action on Research in Health Services (PARIHS) framework. Implement Sci.

[54] McCormack B, McCarthy G, Wright J, Slater P, Coffey A (2009). Development and testing of the Context Assessment Index (CAI). Worldviews Evid Based Nurs.

[55] Hartveit M, Hovlid E, Nordin MHA (2019). Measuring implementation: development of the implementation process assessment tool (IPAT). BMC Health Serv Res.

[56] Rogers EM. Diffusion of Innovations. 5 ed ed. New York: Free Press; 2003.

[57] Rycroft-Malone J, Kitson A, Harvey G (2002). Ingredients for change: revisiting a conceptual framework. Qual Saf Health Care.

[58] Harvey G, Kitson A (2016). PARIHS revisited: from heuristic to integrated framework for the successful implementation of knowledge into practice. Implement Sci.

[59] Squires JE, Graham I, Bashir K (2019). Understanding context: a concept analysis. J Adv Nurs.

[60] Wensing M (2017). The Tailored Implementation in Chronic Diseases (TICD) project: introduction and main findings. Implement Sci.

[61] Squires JE, Aloisio LD, Grimshaw JM (2019). Attributes of context relevant to healthcare professionals’ use of research evidence in clinical practice: a multi-study analysis. Implement Sci.

[62] Goodman LA (1961). Snowball sampling. Ann Math Statist.

[63] Francis JJ, Johnston M, Robertson C (2010). What is an adequate sample size? operationalising data saturation for theory-based interview studies. Psychol Health.

[64] Sibley KM, Roche PL, Bell CP, Temple B, Wittmeier KDM (2017). A descriptive qualitative examination of knowledge translation practice among health researchers in Manitoba, Canada. BMC Health Serv Res.

[65] Kuzel AJ (1999). Sampling in qualitative inquiry.

[66] Squires JE, Graham N, Coughlin M (2018). Barriers and enablers to organ donation after circulatory determination of death: a qualitative study exploring the beliefs of frontline intensive care unit professionals and organ donor coordinators. Transplant Direct.

[67] QSR International Pty Ltd. NVivo Qualitative Data Analysis Software (Version 10). 2012.

[68] Sandelowski M (2000). Whatever happened to qualitative description?. Res Nurs Health.

[69] Hsieh HF, Shannon SE (2005). Three approaches to qualitative content analysis. Qual Health Res.

[70] Benner P, Tanner C, Chesla C. Expertise in Nursing Practice: Caring, Clinical Judgment, and Ethics. New York: Springer Publishing Company; 2009.

[71] Gibbs GR. Analyzing Qualitative Data. London: Sage; 2008.

[72] Lincoln YS, Guba EG (1986). But is it rigorous? trustworthiness and authenticity in naturalistic evaluation. New Directions for Program Evaluation.

[73] Patton MQ (1999). Enhancing the quality and credibility of qualitative analysis. Health Serv Res.

[74] Beidas RS, Stewart RE, Adams DR (2016). A multi-level examination of stakeholder perspectives of implementation of evidence-based practices in a large urban publicly-funded mental health system. Adm Policy Ment Health.

[75] Harvey G, Jas P, Walshe K (2015). Analysing organisational context: case studies on the contribution of absorptive capacity theory to understanding inter-organisational variation in performance improvement. BMJ Qual Saf.

[76] Hinchcliff R, Greenfield D, Westbrook JI, Pawsey M, Mumford V, Braithwaite J (2013). Stakeholder perspectives on implementing accreditation programs: a qualitative study of enabling factors. BMC Health Serv Res.

[77] Holtkamp KC, Vos EM, Rigter T, Lakeman P, Henneman L, Cornel MC (2017). Stakeholder perspectives on the implementation of genetic carrier screening in a changing landscape. BMC Health Serv Res.

[78] Muellmann S, Steenbock B, De Cocker K (2017). Views of policy makers and health promotion professionals on factors facilitating implementation and maintenance of interventions and policies promoting physical activity and healthy eating: results of the DEDIPAC project. BMC Public Health.

[79] Renz AD, Conrad DA, Watts CA (2013). Stakeholder perspectives on the implementation of shared decision making: a qualitative data analysis. Int J Healthc Manag.

[80] Schneider JL, Davis J, Kauffman TL (2016). Stakeholder perspectives on implementing a universal Lynch syndrome screening program: a qualitative study of early barriers and facilitators. Genet Med.

[81] Palinkas LA, Schoenwald SK, Hoagwood K, Landsverk J, Chorpita BF, Weisz JR (2008). An ethnographic study of implementation of evidence-based treatments in child mental health: first steps. Psychiatr Serv.

[82] Hamilton AB, Brunner J, Cain C (2017). Engaging multilevel stakeholders in an implementation trial of evidence-based quality improvement in VA women’s health primary care. Transl Behav Med.

[83] Ritchie MJ, Parker LE, Edlund CN, Kirchner JE (2017). Using implementation facilitation to foster clinical practice quality and adherence to evidence in challenged settings: a qualitative study. BMC Health Serv Res.

[84] Squires JE, Graham ID, Hutchinson AM (2015). Identifying the domains of context important to implementation science: a study protocol. Implement Sci.

